# Swedish translation and validation of the Pediatric Insomnia Severity Index

**DOI:** 10.1186/s12887-020-02150-5

**Published:** 2020-05-26

**Authors:** Charlotte Angelhoff, Peter Johansson, Erland Svensson, Anna Lena Lena Sundell

**Affiliations:** 1 Crown Princess Victoria’s Child and Youth Hospital, and Department of Biomedical and Clinical Sciences, Linköping University, SE-58185 Linköping, Sweden; 2 Department of Health Care Sciences, Palliative Research Centre, Ersta Sköndal Bräcke University College, Stockholm, Sweden; 3 Department of Cardiology and Department of Medical and Health Sciences, Linköping University, Linköping, Sweden; 4 (Retired) Swedish Defense Research Agency, Linköping, Sweden; 5 Department of Pediatric Dentistry, Institute for Postgraduate Dental Education, Jönköping, Sweden; 6 Centre of Oral Health, School of Health Sciences, Jönköping University, Jönköping, Sweden

**Keywords:** Child, Child, preschool, Health promotion, Sleep, Translations, Pediatrics, Validation studies, Quality of life

## Abstract

**Background:**

To increase health and well-being in young children, it is important to acknowledge and promote the child’s sleep behaviour. However, there is a lack of brief, validated sleep screening instruments for children. The aims of the study were to (1) present a Swedish translation of the PISI, (2) examine the factor structure of the Swedish version of PISI, and test the reliability and validity of the PISI factor structure in a sample of healthy children in Sweden.

**Methods:**

The English version of the PISI was translated into Swedish, translated back into English, and agreed upon before use. Parents of healthy 3- to 10-year-old children filled out the Swedish version of the PISI and the generic health-related quality of life instrument KIDSCREEN-27 two times. Exploratory and confirmatory factor analyses for baseline and test-retest, structural equation modelling, and correlations between the PISI and KIDSCREEN-27 were performed.

**Results:**

In total, 160 parents filled out baseline questionnaires (test), whereof 100 parents (63%) filled out the follow-up questionnaires (retest). Confirmative factor analysis of the PISI found two correlated factors: sleep onset problems (SOP) and sleep maintenance problems (SMP). The PISI had substantial construct and test-retest reliability. The PISI factors were related to all KIDSCREEN-27 dimensions.

**Conclusions:**

The Swedish version of the PISI is applicable for screening sleep problems and is a useful aid in dialogues with families about sleep.

## Background

Sleep disturbances in children are an increasing public health problem. One out of four children under the age of five has been reported by their parents to have sleep disturbances [1], leading to physical as well as behavioural problems [1–3]. Sleep is essential for children’s health and is associated with health-related quality of life (HRQoL) [4, 5], which includes children’s well-being and subjective health.

To increase health and well-being in young children, it is important to acknowledge and promote the child’s sleep behaviour. Child health care providers, who regularly meet young children and their parents, play a major role in detecting sleep disturbances in children [6, 7]. However, parental knowledge about the signs and consequences of sleep disturbances in children is poor, and if parents do not recognize when their children’s sleep habits fall outside the expected range for their age, they might not support and encourage the child to practise healthy sleep [8].

Children’s sleep should be considered more seriously in the public health community, and a brief instrument with questions that captures the dimensions of sleep health well, is easy to administer, and is reliable and valid is needed to measure children’s sleep [9]. There is a lack of brief, validated sleep screening instruments for children [7, 8]. However, the Pediatric Insomnia Severity Index (PISI), a brief, 6-item parent-proxy instrument, was constructed, validated and reliability-tested in English for quantifying insomnia symptoms in children 4–10 years old [10]. Parent report of children’s (9–17 years old) sleep has been found to be comparable to objectively measured sleep and thus is appropriate for clinical and research applications [11]. To our knowledge, there is no brief, validated instrument in Swedish for measuring children’s sleep.

The aims of the study were to [1] present a Swedish translation of the PISI, [2] examine the factor structure of the Swedish version of PISI, and test the reliability and validity of the PISI factor structure in a sample of healthy children in Sweden.

## Methods

### Participants and procedure

Parents (*n* = 188) of children 3–10 years old, with no major health problems, were asked to participate in the study when visiting child health care centres in Region Östergötland and public dental health services in Region Jönköping County for regular health visits with their children. After informed consent, the parents received a coded form with instructions and questionnaires. The completed form was placed in a postage-paid envelope and returned to the authors (CA and ALS). Four weeks later, the parents received a new identical form at their home address together with a postage-paid envelope. The parents were contacted via phone by a research assistant if the form was not returned within two weeks, and if needed, once again after another two weeks. Data collection was ongoing between September 2018 and May 2019.

### Questionnaires

#### The Pediatric Insomnia Severity Index (PISI)

The PISI is a 6-item parent-proxy measure designed to monitor primary clinical symptoms of paediatric insomnia for children 4–10 years old, which was developed in the USA. The PISI items follow the International Classification of Sleep Disorders (ICSD-II) general criteria for insomnia (i.e., difficulties falling asleep, difficulties maintaining sleep, and daytime impairment). Items 1–5 are rated on a 6-point scale from “never” (0 points) to “always/7 days a week” (6 points), with a maximum score of 30 points. The total sleep duration (item 6) is rated on a 6-point scale estimating total hours of sleep on most nights, where a lower score indicates more hours of sleep (0 = 11–13 h of sleep and 6 = < 5 h of sleep). The PISI has been reliability and validity tested in children (4–10 years old) with a clinical diagnosis of insomnia at a sleep disorders centre in a paediatric hospital. A two-factor solution was established after removal of item 5 describing daytime sleepiness. The PISI is sensitive and has been validated for brief screening of insomnia symptoms or ongoing assessment during clinical care for paediatric patients. There are currently no empirically established cut-off scores for insomnia diagnosis [10, 12].

#### KIDSCREEN-27

Since there is no brief instrument in Swedish for measuring children’s sleep, we used a generic HRQoL instrument for criterion validity (in reality concurrent validity agreement with the true value - gold standard). We compared the PISI with the validated and reliability-tested proxy version of the HRQoL questionnaire KIDSCREEN-27. KIDSCREEN-27 contains five dimensions of HRQoL: physical well-being (PHY, 5 items), psychological well-being (PWB, 7 items), autonomy and parent relations (PAR, 7 items), social support and peers (SOC, 4 items), and school environment (SCH, 4 items). Each item is scored on a 5-point Likert-type scale (1 = no agreement at all and 5 = total agreement), where higher values indicate better HRQoL, and the maximum score is 100 [13, 14]. A general KIDSCREEN-27 factor was formed by adding up T-values from the 5 dimensions dived with 5. There are no empirically established cut-off scores for low or high HRQoL. Approval for use was obtained from the copyright holders.

### Translation procedure

The process to translate the PISI was approved by Professor Kelly C. Byars of Cincinnati Children’s Hospital in October 2017. The translation was performed according to the guidelines provided by the ISPOR Translation and Cultural Adaptation group [15]. The original English version was translated into Swedish by one of the authors (CA), whose native language was Swedish. This version was discussed and agreed upon (CA and PJ) before the Swedish version was translated back into English by a native English-speaking certified translator. This version was then reviewed by CA and PJ. No conceptual differences were found when comparing the Swedish version to the original English version (Suppl. file).

### Statistics

Descriptive statistics were used to describe the study population and are reported in terms of means and standard deviations (sd) or in frequencies (*n*) and percentages (%).

The construct validity of the Swedish version of the PISI was established by exploratory and confirmatory factor analyses. To explore the factor structure of the six items in the PISI, data collected at baseline, exploratory factor analysis, principal component analysis, and factor analysis with oblique rotation were used. Criteria for the item to be retained in a factor were that they had to achieve a factor loading of at least 0.3. To determine the number of factors, eigenvalues larger than one, scree tree plots, and theory-based selection were used. In order to examine and test the extent to which the data collected could represent the factor model and be generalizable to the population, the final exploratory factor analysis was tested by performing two confirmatory factor analyses, one on data collected at baseline and the second one on data collected at test-retest.

Criterion validity was explored by analysing the association between the factors in the PISI and HRQoL as assessed by KIDSCREEN-27. We assumed that the more problems with sleep, the poorer the HRQoL was [4, 5]. Thus, there should be a negative association between the PISI and KIDSCREEN-27. In the analysis of criterion validity, both correlations and structural equation modelling (SEM) was used to explore the associations of the factors in the PISI to each of the five KIDSCREEN-27- dimensions. It is reasonable to assume that the five KIDSCREEN-27 dimensions are correlated, and that a general KIDSCREEN-27 factor forms an optimal combination of the five dimensions. Accordingly, the relations between this summarizing KIDSCREEN-27 measure and the PISI factors were analysed and modelled. Goodness of fit tests are reported here as the chi-square (χ^2^) value, including degrees of freedom (df), root mean square error of approximation (RMSEA) and comparative fit index (CFI). An overall RMSEA below 0.06 and a confidence interval range from 0.00 to 0.08 indicates a good fit. A CFI value equal or above 0.95 is considered a very good fit [16]. In the SEM analysis, standardized effects found between 0.10 and 0.30 are considered to be small, effects found between 0.30 and 0.50 are considered as moderate, and effects greater than 0.50 are considered to be strong.

Reliability was analysed by construct reliability, indicating to what extent the items in the PISI provide reliable measures of the factors. Values larger than 0.60 are desirable [17]. We also explored reliability by analysing the association between the factors in the PISI at baseline and test-retest.

Descriptive statistics were analysed using SPSS version 25.0. The exploratory and confirmatory factor analyses and SEM analysis were performed with LISREL software [18]. A level of *p* < 0.05 was regarded as statistically significant.

## Results

### Participants

In total, 160 parents filled out baseline questionnaires (test) whereof 100 parents filled out the follow-up questionnaires (retest). The average number of days between test and retest was 64.6 days (sd ± 39.2 days). Seventy percent of the questionnaires were answered by mothers. Mean age for the children was 6.9 years old (sd ± 2.2 years old, range 3.0–10.7 years old). Forty-four percent of the children were girls.

### Exploratory and confirmatory factor analyses

After a series of exploratory factor analyses, we found that the communality (common variance with other variables) of item 6 (hours of night sleep) was low, and accordingly, it was excluded in further analyses. The final exploratory model was found to have two factors: sleep onset problems (SOP) (item 1 “My child takes longer than 30 minutes to fall asleep after going to bed” and item 2 “My child has problem falling asleep at bedtime”) and sleep maintenance problems (SMP) (item 3 “My child awakes more than once during the night”, item 4 “After waking during the night, my child has trouble returning to sleep” and item 5 “My child appears sleepy during the day”). From confirmative factor analyses, which were based on the exploratory factor model, we found that two dimensions are needed to account for the common variance between the five variables of the PISI.

**Fig. 1:**
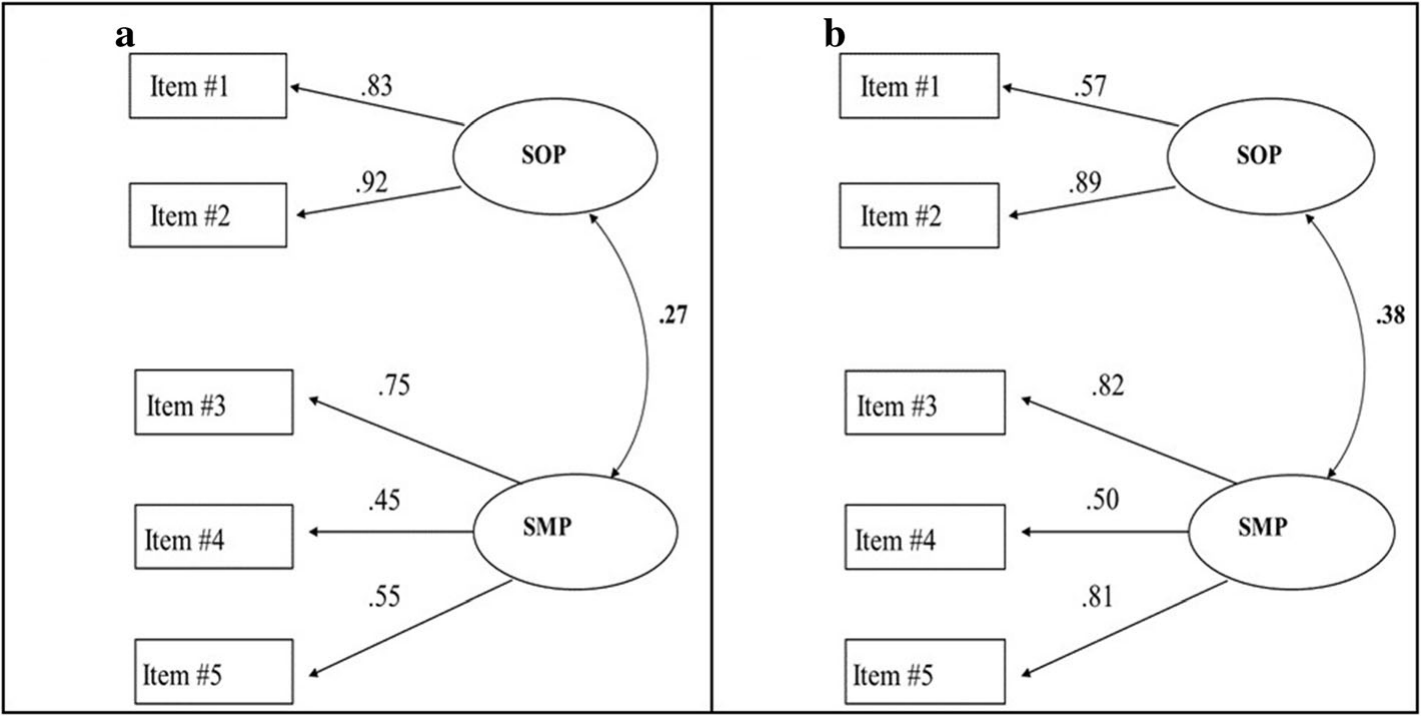
The confirmatory factor analyses of the Swedish version of PISI. **a** presents the confirmatory model for data collected at baseline (test) and **b** presents the model for data collected at re-test. All factor loadings and factor inter-correlations are significant (*p* < 0.05)

Figure 1a and b present the confirmatory analysis two-factor solutions, SOP and SMP, for baseline (test) and follow-up (re-test), respectively. Both models showed a good fit. The fit was χ^2^ = 0.43, df = 3, *p* = 0.93, RMSEA = 0.00, and CFI = 0.99 at baseline, and the fit was χ^2^ = 0.23, df = 2, *p* = 0.89, RMSEA = 0.00, and CFI = 0.99 at test-retest. As can be seen, SOP and SMP are positively correlated (baseline *r* = 0.27, and test-retest *r* = 0.38). The construct reliability for SOP and SMP at baseline was 0.86 and 0.62, respectively. The corresponding value for SOP and SMP at test-retest was 0.71 and 0.76, respectively, indicating that the construct reliability of the Swedish version of the PISI is reliable and replicable.

To further analyse the construct validity and reliability, we explored (using SEM) how the SOP and SMP at baseline was associated with SOP and SMP at retest.

**Fig. 2:**
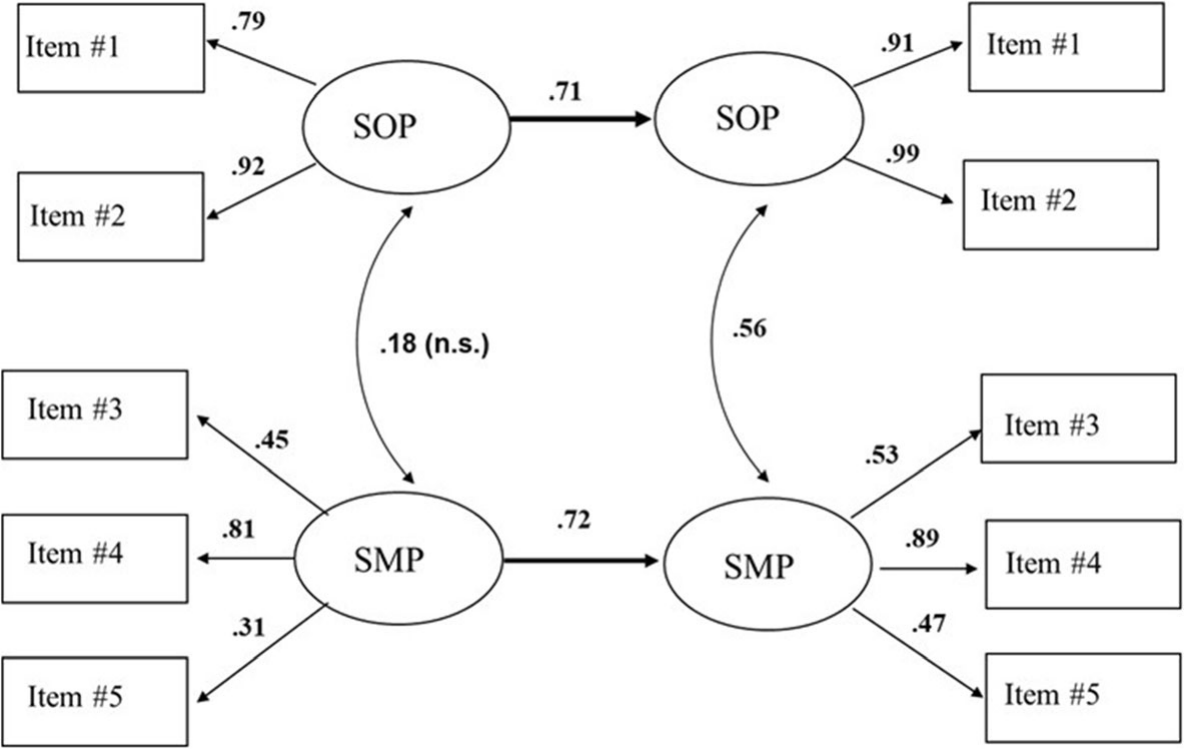
A combined model of the association between the confirmatory models at baseline (to the left), and at test-retest (to the right). Chi-square = 30.20, df = 24, *p* = 0.178, RMSEA = 0.051, CFI = 0.98

Figure 2 shows that the model has a good fit (i.e., χ^2^ = 30.20, df = 24, *p* = 0.18, RMSEA = 0.05, and CFI = 0.98), and SOP and SMP at baseline were highly correlated with SOP and SMP at test-retest (*r* = 0.71 and *r* = 0.72, respectively). Thus, SOP and SMP at baseline have a substantial effect or predictive power on SOP and SMP at test-retest. More than 50% of the true variance in SOP and SMP at test-retest can be explained by the variance of the factors at baseline. The baseline/test-retest correlations also support the reliability of the factors in the PISI.

**Fig. 3:**
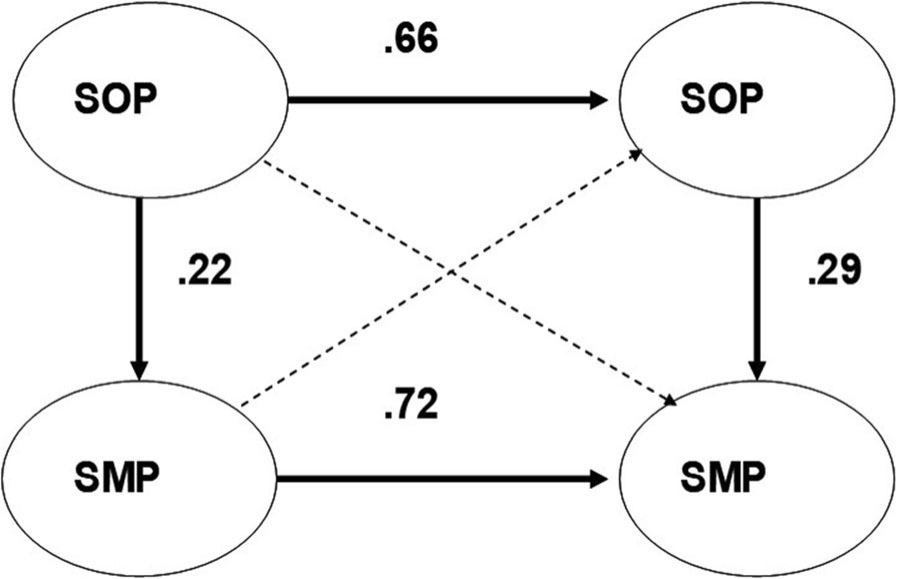
The empirical correlations between the factor-means of sleep onset problems (SOP) and sleep maintenance problems (SMP) at base-line and at re-test, respectively, and the relations SOP and SMP at base-line and at re-test. All correlations, except the dashed cross-lagged relations, are significant (*p* < 0.05)

To make the factors practicable, the means of the variables of each factor in the PISI have been calculated (with equal weight of the variables) and then correlated. As can be seen, the correlations of Fig. 3 are in correspondence (*r* = 0.66 and *r* = 0.72, respectively) with the model in Fig. 2 (*r* = 0.71 and *r* = 0.72, respectively).

It is possible that the child’s age may influence the parent’s response in the PISI. Therefore, we performed a re-analysis on the model in Fig. 2 and controlled for age by means of partial correlation analysis. The results showed that the model was stable, thus the age of the children had no influence on the model.

Taken all together, this indicates that the two-dimensional structure of the Swedish version of the PISI has substantial construct and test-retest reliability.

### Criterion validity of the PISI and KIDSCREEN-27

To explore the criterion validity of the PISI, we analysed the correlations between the two factors in the PISI (SOP and SMP) from the baseline measurement and test-retest measurements to the five dimensions in KIDSCREEN-27. The correlations were optimized by means of confirmative factor analyses. The correlations between SOP and SMP from the two data collection points and the five dimensions in KIDSCREEN-27 were generally weak and non-significant for SOP (Table 1). But, SMP, on the other hand, correlated significantly with all dimensions in KIDSCREEN-27. However, SOP and SMP are correlated, and it can be reasonable to assume that the former affects the latter, and problems with falling asleep in the evening (i.e., SOP) may cause sleeping problems during the night (i.e., SMP) (Fig. 1). Therefore, we performed a series of SEM analyses using SOP and SMP.

**Table 1:** Optimally weighted correlations^a^ between SOP and SMP and the five criterion dimensions of KIDSCREEN-27

		School environment		Psychological well-being		Autonomy and parent relations		Social support and peers		Physical well-being	
		*r*	*p-*value	*r*	*p-*value	*r*	*p-*value	*r*	*p-*value	*r*	*p-*value
Sleep											
Onset	Test	−.07	.38	−.17*	.03	−.14	.09	−.16*	.04	−.06	.46
Problems	ReTest	−.10	.35	−.17	.10	−.04	.71	−.02	.85	.13	.22
Sleep											
Maintenance	Test	−.40*	<.01	−.36*	<.01	−.10	.23	−.23*	.04	−.31*	<.01
Problems	ReTest	−.47*	<.01	−.48*	< .01	.52*	<.01	−.26*	.01	−.42*	<.01

^a^ Pearson correlation coefficient (*r*)

* Significant correlations (*p <* .05)

**Fig. 4:**
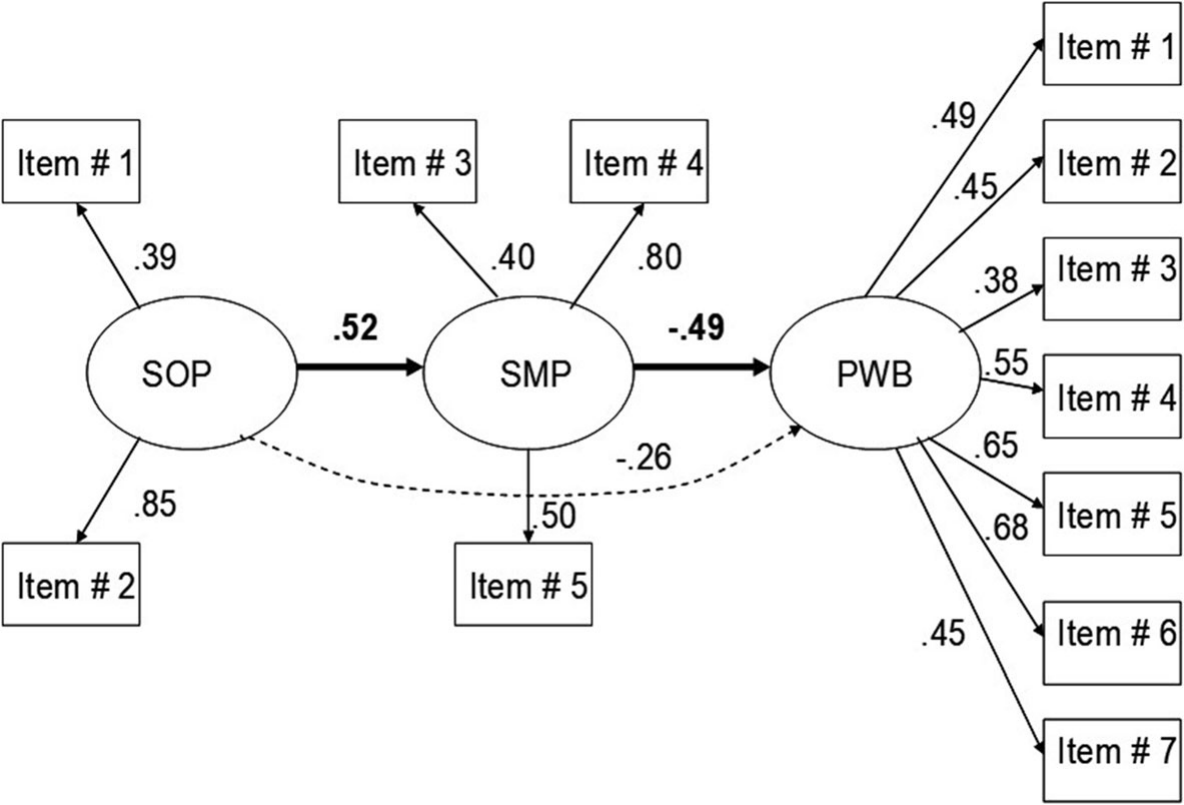
Structural equation model (SEM) of the factors sleep onset problems (SOP), sleep maintenance problems (SMP), and psychological wellbeing (PWB). Chi- square = 51.83, df = 41, *p* = 0.190, RMSEA = 0.044, CFI = 0.97. All effects and factor-loadings are significant (*p* < 0.05)

Table 2 presents the indirect and direct effects from SOP and SMP on the dimensions of KIDSCREEN-27. As can be seen, SOP and SMP had effects on all dimensions of the KIDSCREEN-27. The models showed that there were significant direct effects of SMP on the criterion measures and significant indirect effects of SOP on the criterion measures. However, in the SOC dimension, no significant indirect effect of SOP could be found. The predictive power (i.e., the ability to “explain” the variance of the criterion dimensions) of the two factors ranged from 7 to 27% (7% for SOC, 18% for PHY, 22% for SCH, 23% for PWB, and 27% for PAR). Figure 4 shows the model for PWB as an example of the analyses. The model had a good fit (χ^2^ = 51.83, df = 41, *p* = 0.19, RMSEA = 0.04, and CFI = 0.97) and showed that SMP has a direct effect (B = − 0.49), indicating a decreasing effect on PWB. For SOP, there was a direct effect (B = 0.52) on SMP, indicating that SOP increases SMP, and also an indirect negative effect on PWB (B = − 0.26), indicating that SMP is a mediating factor between SOP and PWB.

**Table 2:** Correlations between the PISI and KIDSCREEN-27

Criterion-dimension “Re-test”	Physical well-being (PHY)		Autonomy and parent relations (PAR)		Social support and peers (SOC)		School environment (SCH)		Psychological well-being (PWB)	
Effects “Re-Test”	Indirect Effect	Direct Effect	Indirect Effect	Direct Effect	Indirect Effect	Direct Effect	Indirect Effect	Direct Effect	Indirect Effect	Direct Effect
SOP “Re-Test”	−.14		−.26		−.19 (n.s.)		−.21		−.26	
SMP “Re-Test”		−.32		−.47		−.19		−.45		−.49
Model fit- indices										
Chi-square	38.06		56.45		29.00		24.21		51.83	
df	30		44		25		22		41	
P	.15		.10		.25		.37		.19	
RMSA	.05		.05		.04		.03		.04	
CFI	.97		.97		.98		.98		.97	

Direct and indirect effects from structural equation models of the factors sleep onset problems (SOP), and sleep maintenance problems (SMP) on the KIDSCREEN-27 domains Physical well-being, Autonomy and parent relations, Social support and peers, School environment and Psychological wellbeing. The figures in the table are based on the models from the re-test-situation. n.s = non-significant

When scrutinizing the KIDSCREEN-27-dimensions, we found a mean correlation between the five dimensions of .44, and accordingly, a “second order factor” was to be expected. In a confirmative second order factor analysis, we found that the five dimensions formed a second order general KIDSCREEN-27 factor, and SMP (test-retest) has a factor loading of −.48 with the general KIDSCREEN-27 factor (χ^2^ = 8.42, df = 9, *p* = 0.49, RMSEA = 0.00, CFI = 0.99). Thus, the SMP dimension is directly or indirectly related to all five KIDSCREEN-27 factors and explains 23% of the variance of the general KIDSCREEN-27 factor. The general KIDSCREEN-27 factor represents an optimally weighted combination of the five KIDSCREEN-27 dimensions.

## Discussion

In the present study, the PISI was translated into Swedish. Reliability and validity was tested in healthy children 3–10 years old as compared to Byars et al. [10] who tested the PISI in a population of children with a clinical diagnosis of insomnia at a sleep disorders centre. In both studies, the PISI was found to be well suited for assessment of children’s sleep despite different populations (i.e., children diagnosed with insomnia/healthy children and children with different nationalities).

From confirmative factor analyses, we found that two correlated factors, SOP and SMP, were needed in order to explain the co-variances between the variables of the instrument. These results are in line with the results from Byars et al. [10]. The construct reliabilities (indicating to what extent the markers provide reliable measures of the construct or factor) were larger than 0.60, which indicate good reliability [17]. What this study adds is that the test-retest reliabilities of the two factors were high, indicating that about 50% of the variance of the re-test was explained by the baseline test. Accordingly, the items of the PISI are reliable measures of SOP and SMP.

We assumed that problems with falling asleep in the evening (SOP) caused sleeping problems during the night (SMP), and the time factor supports this assumption. This conclusion in combination with our findings that only SMP is directly related to the KIDSCREEN-27 dimensions formed the basis for the model in which SOP associates to SMP, and SMP, in turn, associates to the KIDSCREEN-27 dimensions. However, SOP could be underestimated if parents compensated their child’s sleep onset difficulties by being present near the child until they fall asleep. The child may then have SMP after waking, finding the parent absent.

Of special interest is that significant indirect effects were also found between SOP and the KIDSCREEN-27 dimensions. These indirect effects clearly indicate that SMP acts as a mediator, and without this factor, no effects of SOP on the KIDSCREEN-27 dimensions have been found. The model represents a simplex structure or quasi-Markov chain (a sequence in which each event is dependent on the state in the previous events), which often has been found to represent psychological processes.

The Swedish version of the PISI explains a substantial proportion of the true variance of the criterion dimensions and has effective and practicable criterion validity with respect to its short number of items in comparison to the number of items in KIDSCREEN-27. It is also of interest to note that the PISI factors is related to all five KIDSCREEN-27 dimensions. A conclusion could be that the PISI factors represent sleep problems of general importance for most areas of functioning. The strong correlation between the PISI and the second order factor of KIDSCREEN-27 supports the PISI’s relationship to HRQoL.

In the present study, we found strong correlations between sleep and HRQoL. There are few studies of sleep and HRQoL in young children. An Australian study reported that sleep quality predicted HRQoL in children 10–11 years old [19]. In Finland, Gustafsson et al. [4] found an association between sleep duration and HRQoL in children 10–15 years old. Contradictory results were found by Price et al. [20], who showed weak and inconsistent correlations between sleep duration and HRQoL in Australian children 4–9 years old. However, none of these studies used a validated sleep assessment tool. Moreover, an American study found associations between insomnia and HRQoL in children 7–10 years old, using ICSD-II [5]. More research using a validated sleep assessment tool is needed to get more knowledge about sleep in healthy children and its correlation to HRQoL.

A strength of this study is that there was a high response rate, as 63% of the parents completed the questionnaires twice. Healthy children from different contexts (e.g., child care centres and public dental clinics) from different counties were included, and the proportion of girls vs. boys was nearly 1:1. This suggests that our results could be generalized in healthy children in other clinic settings or county samples. However, there are some study limitations that need to be considered. The number of days between the test and re-test were longer than planned, an average of 2 months. On the other hand, this did not seem to have any effect on the results since SOP and SMP at baseline were highly correlated with SOP and SMP at test-re-test. Another limitation is that only parents of healthy children or children with minor health problems were included in the study. The Swedish version of the PISI has not been validated in children with major health problems. The ability to differentiate children with and without sleep problems was not assessed as the sample only included healthy children and not children with known insomnia or other sleep disorders. As the PISI is answered by proxy and the items are developed from ICSD-II criteria for insomnia, we suggest that the PISI could even be used in other groups of children.

Considering the high prevalence of sleep disturbances in young children, there is a need to acknowledge and promote sleep in children. A lack of brief instruments to measure children’s sleep may make it difficult for health care professionals to determine sleep problems in young children. To counteract the negative effects of insufficient sleep, a public health policy to promote sleep health in the paediatric population is essential [9]. The PISI could be used in a dialogue about the child’s sleep during health care visits in primary health care centres as well as other contexts, such as dentistry and school. Moreover, the PISI is a brief measurement for research in both healthy children and children with poor sleep. Further investigations of critical values of the PISI to find a cut-off score could be helpful for symptom screening and future research studies of sleep in children.

## Conclusion

The Swedish version of the PISI, as a proxy report instrument, appears to be reliable and valid for identifying sleep problems in healthy children and can aid in dialogues with families about sleep. Further research is needed for its ability to detect sleep disorders and improvements following treatment.

## Supplementary information

**Additional file 1.**

## Data Availability

The datasets used and analysed during the current study are available from the corresponding author on reasonable request.

## Abbreviations

CFI: Confirmatory Fit Index
PAR: Autonomy and parent relations
PISI: Pediatric Insomnia Severity Index
PHY: Physical well-being
PWB: Psychological well-being
RMSEA: The Root Mean Square Error of Approximation
SCH: School environment
SEM: Structural equation modelling
SOC: Social support and peers
SOP: Sleep onset problems
SMP: Sleep maintenance problems
