# Correction to: Swedish translation and validation of the Pediatric Insomnia Severity Index

**DOI:** 10.1186/s12887-020-02232-4

**Published:** 2020-07-04

**Authors:** Charlotte Angelhoff, Peter Johansson, Erland Svensson, Anna Lena Sundell

**Affiliations:** 1 Crown Princess Victoria’s Child and Youth Hospital, and Department of Biomedical and Clinical Sciences, Linköping University, SE-58185 Linköping, Sweden; 2 Department of Health Care Sciences, Palliative Research Centre, Ersta Sköndal Bräcke University College, Stockholm, Sweden; 3 Department of Cardiology and Department of Medical and Health Sciences, Linköping University, Linköping, Sweden; 4 (Retired) Swedish Defense Research Agency, Linköping, Sweden; 5 Department of Pediatric Dentistry, Institute for Postgraduate Dental Education, Jönköping, Sweden; 6 Centre of Oral Health, School of Health Sciences, Jönköping University, Jönköping, Sweden

**Correction to: BMC Pediatrics (2020) 20:253**

**https://doi.org/10.1186/s12887-020-02150-5**

Following the publication of [1], there was error in capturing the author name in the published online version. Anna Lena Sundell was captured as Anna Lena Lena Sundell. Correct name is shown above.

The original article has been corrected.
