# Supplementary material for: Swedish translation and validation of the Pediatric Insomnia Severity Index
Source: BMC Pediatr. 2020 May 26;20:253. doi: 10.1186/s12887-020-02150-5 (PMC7249377; doi:10.1186/s12887-020-02150-5)
Supplement: Supplementary file 1 — Additional file 1. [file 12887_2020_2150_MOESM1_ESM.pdf]

Pediatric Insomnia Severity Index

Please answer each of the following questions about your child's sleep. The following statements are about your child's sleep habits and possible difficulties with sleep. Think about the <u>past week</u> in your child's life when answering these questions. If last week was unusual for a specific reason (such as your child had an ear infection and did not sleep well or the TV set was broken), choose the most recent typical week.

Use the following scale when answering the questions

	0	1	2	3	4	5
	**Never**	**Once in a while**	**Sometimes**	**Quite Often**	**Frequently**	**Always**
	(0 nights)	(1-2 nights)	(2-3 nights)	(4-5 nights)	(5-6 nights)	(7 nights)
1) My child takes longer than 30 minutes to fall asleep after going to bed	0	1	2	3	4	5
2) My child has trouble falling asleep at bedtime.	0	1	2	3	4	5
3) My child awakes more than once during the night	0	1	2	3	4	5
4) After waking during the night may child has trouble returning to sleep	0	1	2	3	4	5
5) My child appears sleepy during the day	0	1	2	3	4	5
	**Number of hours per night**					
	**11-13**	**9-11**	**8-9**	**7-8**	**5-7**	**less than 5**
6) How many hours of sleep does your child get on most nights?	0	1	2	3	4	5

Pediatric Insomnia Severity Index

Var vänlig och svara på var och en av följande frågor om ditt barns sömn. Följande påståenden handlar om ditt barns sömnvanor och eventuella sömnsvårigheter. Tänk på den <u>senaste veckan</u> i ditt barns liv när du svarar på dessa frågor. Om den förra veckan var ovanlig av en särskild anledning (till exempel att ditt barn hade en öroninfektion och inte sov gott eller att tv:n var trasig) välj den senaste typiska veckan.

Använd nedanstående skala när du svarar på frågorna.

	0	1	2	3	4	5
	**Aldrig**	**Då och då**	**Ibland**	**Ganska ofta**	**Ofta**	**Alltid**
	(0 nätter)	(1–2 nätter)	(2–3 nätter)	(4–5 nätter)	(5–6 nätter)	(7 nätter)
1. Det tar mer än 30 minuter för mitt barn att somna på kvällen efter att ha gått till sängs	0	1	2	3	4	5
2. Mitt barn har svårt att somna vid läggdags	0	1	2	3	4	5
3. Mitt barn vaknar mer än en gång på natten	0	1	2	3	4	5
4. Efter att ha vaknat på natten har mitt barn svårt att somna om	0	1	2	3	4	5
5. Mitt barn verkar sömnigt på dagen	0	1	2	3	4	5
			**Antal timmar per natt**			
	**11–13**	**9–11**	**8–9**	**7–8**	**5–7**	**Mindre än 5**
6. Hur många timmars sömn får ditt barn de flesta nätter?	0	1	2	3	4	5
